# Shifts in soil nutrient concentrations and C:N:P stoichiometry during long-term natural vegetation restoration

**DOI:** 10.7717/peerj.8382

**Published:** 2020-01-22

**Authors:** Rentian Ma, Feinan Hu, Jingfang Liu, Chunli Wang, Zilong Wang, Gang Liu, Shiwei Zhao

**Affiliations:** 1 State Key Laboratory of Soil Erosion and Dryland Farming on the Loess Plateau, College of Natural Resources and Environment, Northwest A&F University, Yangling, Shaanxi, China; 2 Institute of Soil and Water Conservation, Chinese Academy of Sciences and Ministry of Water Resources, Yangling, Shaanxi, China

**Keywords:** Vegetation restoration, Soil organic carbon, Nitrogen, Phosphorus, Secondary forest

## Abstract

**Background:**

Ecological stoichiometry (C:N:P ratios) in soil is an important indicator of the elemental balance in ecological interactions and processes. Long-term natural vegetation plays an important role in the accumulation and distribution of soil stoichiometry. However, information about the effects of long-term secondary forest succession on soil stoichiometry along a deep soil profile is still limited.

**Methods:**

We selected Ziwuling secondary succession forest developed from farmland as the study area, investigated the concentrations and stoichiometry of soil organic carbon (SOC), total nitrogen (TN), and total phosphorus (TP) at a depth of 0–100 cm along a 90-year succession chronosequence, including farmland (control), grassland, shrub, early forest, and climax forest.

**Results:**

SOC and TN concentrations significantly increased with increasing restoration age, whereas soil P concentration remained relatively stable across various successional stages. SOC and TN concentrations decreased with an increase in soil depth, exhibiting distinct soil nutrient “surface-aggregation” (high nutrients concentration in the top soil layer). The soil C:P and N:P ratios increased with an increase in restoration age, whereas the variation of the C:N ratio was small and relatively stable across vegetation succession. The nutrient limitation changed along with vegetation succession, transitioning from limited N in the earlier successional stages to limited P in the later successional stages.

**Conclusion:**

Our results suggest that more nitrogen input should be applied to earlier succession stages, and more phosphorus input should be utilized in later succession stages in order to address limited availability of these elements. In general, natural vegetation restoration was an ecologically beneficial practice for the recovery of degraded soils in this area. The findings of this study strengthen our understanding of the changes of soil nutrient concentration and nutrient limitation after vegetation restoration, and provide a simple guideline for future vegetation restoration and reconstruction efforts on the Loess Plateau.

## Introduction

Natural vegetation restoration has been widely used to prevent soil degradation, improve the ecological environment, and rehabilitate degraded environments (Mcgroddy, Daufresne & Hedin, 2004; Grünzweig et al., 2007; Wang et al., 2014). Vegetation restoration can stimulate soil nutrient cycling and maintain soil quality by altering plant species and community composition, litter quality and quantity (Cao & Chen, 2017; Li et al., 2016; Zhao et al., 2015), root architecture and exudates (Berger, Neubauer & Glatzel, 2002; Clemmensen et al., 2013; Deng et al., 2018), and microbial activity (Gao et al., 2014; Gispert et al., 2013). The study of the vegetation restoration process is critical for understanding the relationship between vegetation succession and the changes of soil ecological function, providing a guideline for ecological environment reconstruction or restoration.

In the vegetation restoration process, changes inevitably occur in the composition of soil nutrient elements, particularly in the three main elements: carbon (C), nitrogen (N), and phosphorus (P) (Wei et al., 2009; Zhao et al., 2015). C, N, and P are the three primary nutrients in soils that most influence ecosystem structure and function (Agren, 2008; Tian et al., 2010). To some extent, these elements’ synergistic effects control ecological processes such as the biological elemental cycle and energy transfer (Agren, 2008; Ladanai, Agren & Olsson, 2010; Laik et al., 2009). C:N:P stoichiometry mainly focuses on the interaction and balance of chemical elements in ecological processes (Agren, 2008; Mcgroddy, Daufresne & Hedin, 2004; Ren et al., 2016; Wardle et al., 2004), and provides a useful and effective way to study the distribution, nutrient limitation, and regulatory mechanism of nutrient composition in the ecosystem (Wang et al., 2014). In recent years, many researchers have reported the C:N:P stoichiometry patterns in plant organs (Bai et al., 2019; Yang, Liu & An, 2018), plant communities (Fu et al., 2010; Jiao et al., 2013; Zhao et al., 2017), stand ages (Zhang et al., 2019) and plantations and natural secondary forests (Cao & Chen, 2017; Deng et al., 2016). For example, Yang, Liu & An (2018) found that the C:N:P stoichiometry in leaves, roots, litter, and soil varied hugely, and that the plant community makeup had a significant effect on C:N:P stoichiometry. Li et al. (2019) indicated that natural vegetation restoration increased microbial C:P and N:P ratios in farmland after abandonment. Cao & Chen (2017) documented that natural forest had a greater capacity for C storage than plantation. Despite numerous studies looking at the C:N:P stoichiometry in terrestrial ecosystems (Agren, 2008; Han et al., 2005), C:N:P stoichiometry in soils is yet to be fully described (Manzoni & Porporato, 2009; Zhao et al., 2017), especially in the Loess Plateau of China.

The Loess Plateau is an ecologically fragile area in China. Owing to long-term unsustainable land use and large-scale cultivation of sloping croplands, native vegetation was destroyed, and soil erosion was aggravated in this area (Deng et al., 2016; Fu et al., 2010; Wang, Zhang & Haung, 2009). In order to improve the degraded land, the Chinese government has taken a variety of measures to prevent further soil and water loss and restore the ecosystem (Fu et al., 2000). So far, a large number of croplands have been converted to natural grassland or forest in the Loess Plateau. Soil nutrient concentrations generally undergo dynamic changes over time, and these changes directly influence elemental nutrient stoichiometry (Fan et al., 2015; Ma et al., 2019; Wang et al., 2014). Many studies have found that vegetation restoration enhances soil nutrient status, improves microbial activity (Fan et al., 2015; Ren et al., 2016; Zinn, Marrenjo & Silva, 2018), and improves the C:N:P stoichiometry (Bai et al., 2019; Yang & Luo, 2011). However, other studies have shown that land use change decreases soil nutrient concentrations (Yang et al., 2012), and did not change soil C:N ratio (Zinn, Marrenjo & Silva, 2018). In addition, studies about the effects of vegetation restoration on soil nutrient concentrations and C:N:P stoichiometry are mainly focused on topsoil (0–20 cm), as the topsoil is the source of the majority of plant nutrients and is most vulnerable to human disturbances (Wang, Zhang & Haung, 2009). Recent studies have suggested that the nutrient status of deep soil may also be affected by long-term vegetation restoration (Li et al., 2016; Vandenbygaart et al., 2011). Zhao et al. (2015) demonstrated that long-term afforestation could greatly affect C and N stocks and stoichiometry in deep soil. However, knowledge about the nutrient dynamics of deep soil profiles at various succession stages in arid and semi-arid areas is still lacking, which puts constraints on our ability to understand the geochemical cycles of nutrient elements in these environments. Therefore, we chose the Ziwuling secondary succession forest as the study area, which is developed from farmland and has an intact series of naturally recovering vegetation succession. The concentrations and stoichiometry of SOC, TN, and TP at a depth of 0–100 cm along a 90-year succession chronosequence in this area were investigated. The aim of this study was to: (1) examine the dynamics in soil nutrient concentrations along a long-term natural vegetation succession, and the relationship between soil nutrient content and a 0–100 cm soil profile in a secondary forest region; (2) illustrate the effects of vegetation succession on soil C:N:P stoichiometry and determine nutrient limitations.

## Materials and Methods

### Site description

**Figure 1:**
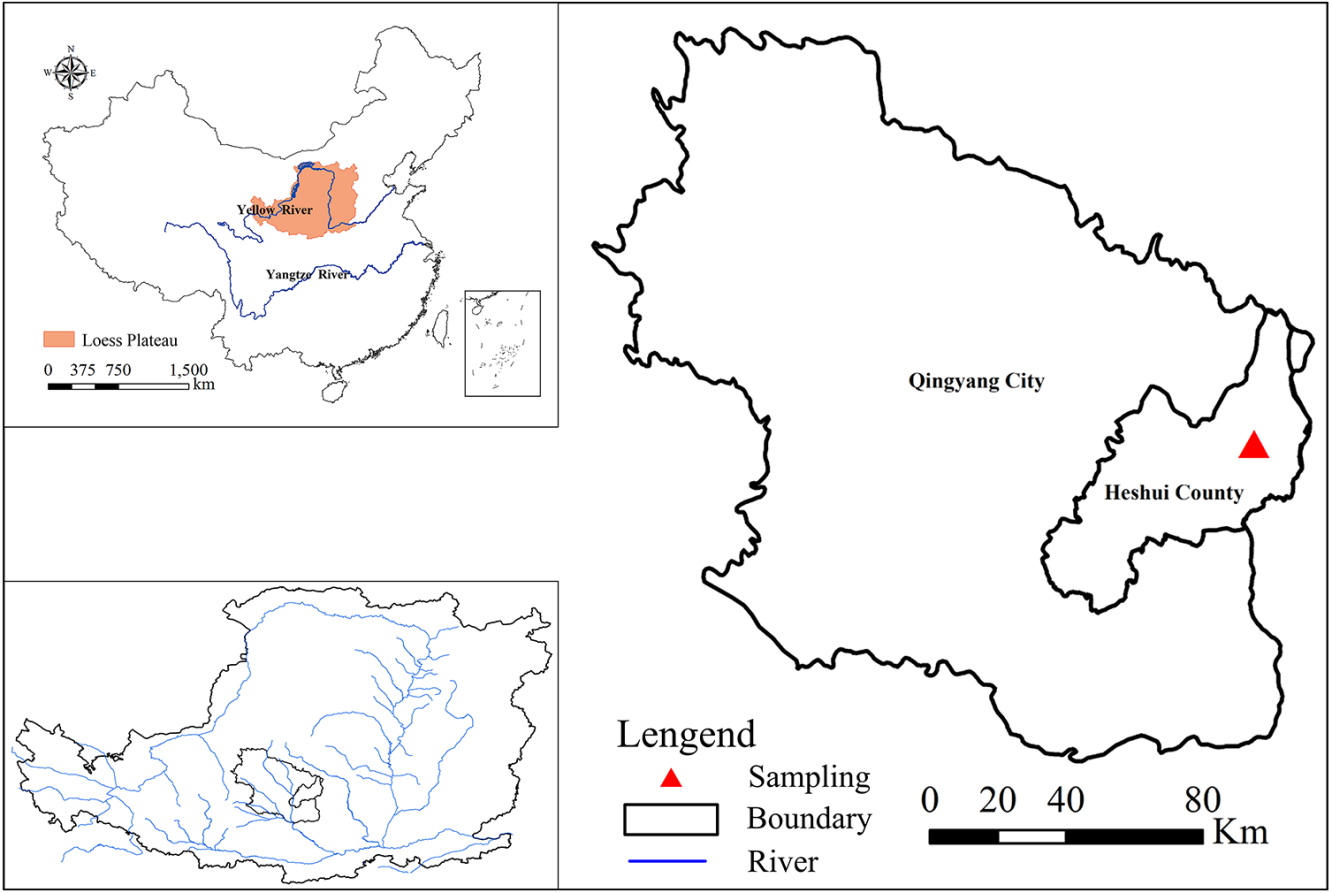
Location of the study site in the Loess Plateau.

The study region is located in Lianjiabian forest farm, Heshui County, Gansu Province, to the north of the Ziwuling Mountains in the Loess Plateau of China (35°03′–36°37′N, 108°10′–109°08′E; altitude 1,245–1,285 m) (Fig. 1). The climate is semi-arid monsoon, with a mean annual rainfall of 587 mm and a mean annual temperature of 7.4 °C. The soil in the study region is typical loessal, developed from native (hillside) or secondary (valley) loess (Jia et al., 2005). The soil is uniformly distributed from a depth of 50 to 130 m, above red earth. At present, the Ziwuling forest area is the most intact natural secondary forest in the Loess Plateau. During the national conflict from 1842 to 1866, many local people were displaced, and secondary forests were established on the abandoned land. Owing to the implementation of “Grain-for-Green Project” in 2000, a large number of farmlands have gradually been abandoned. In the same region, a series of succession stages have formed, along with different restoration ages. Without human interference, the vegetation succession here would progress as follows: grassland (*Bothriochloa ischaemum, Carex lanceolata, Glycyrrhiza*, and *Stipa bungeana* are the main herb species), to shrubland (*Sophora davidii*, *Hippophae rhamnoides*, and *Spiraea pubescens* are the main shrub species), to early forest (*Populus davidiana* and *Betula platyphylla*), to climax forest (*Quercus liaotungensis*) (Cheng et al., 2012).

### Experiment design and soil sampling

After we obtained oral permission from the administrator (Mr. Jifan Wang, the head of Ziwuling Forestry Bureau), we collected the soil samples in mid-June of 2018. According to the vegetation restoration age, structure, and community composition, the typical vegetation type for each stage of the vegetation succession was selected using space-for-time substitution (Zhao et al., 2015), such as *Bothriochloa ischaemum* (Bi) for grassland, *Hippophae rhamnoides* (Hr) for shrubland, *Betula platyphylla* (Bp) and *Populus davidiana* (Pd) for early forest, and *Quercus liaotungensis* (Ql) as climax forest, with adjacent farmland (Fa) as control. The main crop of the farmlands was corn. Corn cultivation followed the traditional cropland practice, farmers plowing the soil at least twice before the crop growing season and applying chemical fertilizers (about 70 kg N ha^−1^ and 23 kg P ha^−1^) twice per year at sowing time and in mid-July. The stand ages of all vegetation types were determined with the help of the local forest management bureau, and confirmed using the hole-boring technique. Three sampling sites were selected as replicates for each vegetation type, and three plots (20 m × 20 m for forest community, 10 m × 10 m for shrub community, and 1 m × 1 m for herbaceous community and farmland) were randomly established within each sampling site. As soils below 100 cm in depth were mixed with small pieces of stones in most sampling sites, we selected 0–100 cm as our sampling depth. After removing leaf litter and humus layer from the soil surface, undisturbed soils were sampled at depths of 0–10, 10–20, 20–40, 40–70, and 70–100 cm using a shovel in each plot. Soil samples of the same layer were mixed into one sampling bag at each sampling site. In total, 90 soil samples (six vegetation types × three sites × five soil layers) were collected. All samples were air-dried, roots and stones were removed, and the sample was passed through a 0.25 mm sieve for SOC, TN, and TP analyses. Table 1 shows the basic information regarding vegetation types, physiographical conditions, and elevation.

**Table 1:** Geographical and vegetation characteristics at different successional stages in the Ziwuling forest region of the Loess Plateau. Bi, Hr, Bp, Pd, Ql, NE and NW represent *Bothriochloa ischemum*, *Hippophae rhamnoides*, *Betula platyphylla*, *Populus davidiana*, *Pinus tabuliformis*, and *Quercus liaotungensis*, northeast and northwest, respectively.

Successional stage	Vegetation types	Restoration ages (year)	Longitude (E)	Latitude (N)	Altitude (m)	Slope (°)	Aspect (°)	Coverage (%)	Other major plant species
Farmland	Corn field	0	108°32′5.99″	36°4′36.20″	1,021	0	NE10	0	–
Grassland	Bi	20	108°31′36.70″	36°5′3.13″	1,345	13	NW74	95	*Stipa grandis*, *Glycyrrhiza uralensis*, *Artemisia giraldii*, and *Carex lanceolata*
Shrub	Hr	30	108°31′36.02″	36°5′5.81″	1,349	10	NW30	70	*Stipa grandis* and *Carex lanceolata*
Early forest	Bp	45	108°32′5.07″	36°4′8.57″	1,339	20	NE2	58	*Carex lanceolata*
	Pd	60	108°31′45.04″	36°2′54.57″	1,449	12	NW13	68	*Spiraea pubescens, Cotoneaster acutifolius, Rosa hugonis*, and *Carex lanceolata*
Climax forest	Ql	90	108°32′32.37″	36°3′5.26″	1,432	17°	NE51°	75	*Spiraea pubescens, Cotoneaster acutifolius, Stipa grandis*, and *Carex lanceolata*

### Soil sample analysis

Soil organic carbon (SOC) was determined by the H_2_SO_4_-K_2_Cr_2_O_7_ oxidation method (Nelson & Sommers, 1982). The 0.5 g soil samples were broken down with 5 ml of 1 M K_2_Cr_2_O_7_ and 5 ml of concentrated H_2_SO_4_ at 180 °C for 5 min, then titrated with standardized FeSO_4_. Total soil N (TN) concentration was measured using the Kjeldahl method, after extraction with sulfuric acid (Sparks et al., 1996). Total soil P (TP) was determined colorimetrically after the sample was broken down with H_2_SO_4_ and HClO_4_ (Parkinson & Allen, 1975).

### Data analysis

Statistical analyses were performed using the Statistical Package for the Social Sciences (SPSS version 20.0 for Windows). Before applying parametric tests, we tested for the normality and homogeneity of the variances. Nested ANOVA was used to systemically test the effect of vegetation types and soil depths on soil nutrient concentrations and stoichiometry. Least Squares Difference (LSD) post hoc tests were used for multiple comparisons. Correlations between C, N, and P concentrations in soil were determined by Pearson’s correlation test at 95% confidence interval. A *P* value of less than 0.05 was considered statistically significant. The C:N, C:P, and N:P ratios of the soil from different vegetation types were computed as mass ratios.

## Results

### SOC, TN, and TP

#### Basic characteristics of SOC, TN, and TP concentrations in soil profile

The SOC, TN, and TP concentrations decreased with an increase in soil depth in the study site. The SOC, TN, and TP concentrations in surface soil (0–20 cm) is high, exhibiting remarkable soil nutrient “surface-aggregation” (high nutrients in the surface soil layer) (Yang, Liu & An, 2018) (Table 2). The SOC concentrations varied greatly from soil depths of 0–10 to 20–40 cm, with the mean concentration ranging from 27.20 to 6.25 g kg^−1^ (decreased of 77.02%). The TN concentrations varied remarkably from soil depths of 0–10 to 20–40 cm, with the average concentration decreasing from 2.39 to 0.68 g kg^−1^ (decrease of 71.55%). The mean concentration of TP from soil depths of 0–10 to 20–40 cm decreased from 0.71 to 0.62 g kg^−1^ (decrease of 12.68%). The coefficient variation (CV) is the main index used to describe the degree of spatial variability of the variables. The SOC and TN concentrations had the highest variability at 0–10 cm with CVs of 0.41 and 0.40, respectively. The variation of TP concentration was smallest, with CV values between 0.09 and 0.11.

**Table 2:** Concentrations of SOC, TN, and TP in soil profile. Means with different lowercase letters were significantly different at the 0.05 level for different soil layers in the same nutrient elements. The sample size *n* = 3.

	Soil depth (cm)	Maximum (g kg^−1^)	Minimum (g kg^−1^)	Mean (g kg^−1^)	Standard deviation (g kg^−1^)	Coefficient of variation
SOC	0–10	40.20	7.20	27.20a	10.42	0.41
	10–20	16.93	6.42	11.12b	2.95	0.27
	20–40	9.14	4.23	6.25c	1.33	0.21
	40–70	6.41	2.67	4.08c	0.92	0.22
	70–100	5.48	2.57	3.37c	0.67	0.20
TN	0–10	3.82	0.98	2.39a	0.95	0.40
	10–20	1.56	0.85	1.11b	0.18	0.16
	20–40	0.94	0.37	0.68c	0.19	0.28
	40–70	0.71	0.25	0.47c	0.15	0.32
	70–100	0.63	0.22	0.39c	0.13	0.32
TP	0–10	0.82	0.60	0.71a	0.06	0.09
	10–20	0.76	0.53	0.66b	0.07	0.11
	20–40	0.71	0.51	0.62c	0.07	0.11
	40–70	0.69	0.54	0.61c	0.06	0.09
	70–100	0.72	0.51	0.61c	0.07	0.11

### Changes of SOC, TN, and TP concentrations in different vegetation types

**Figure 2:**
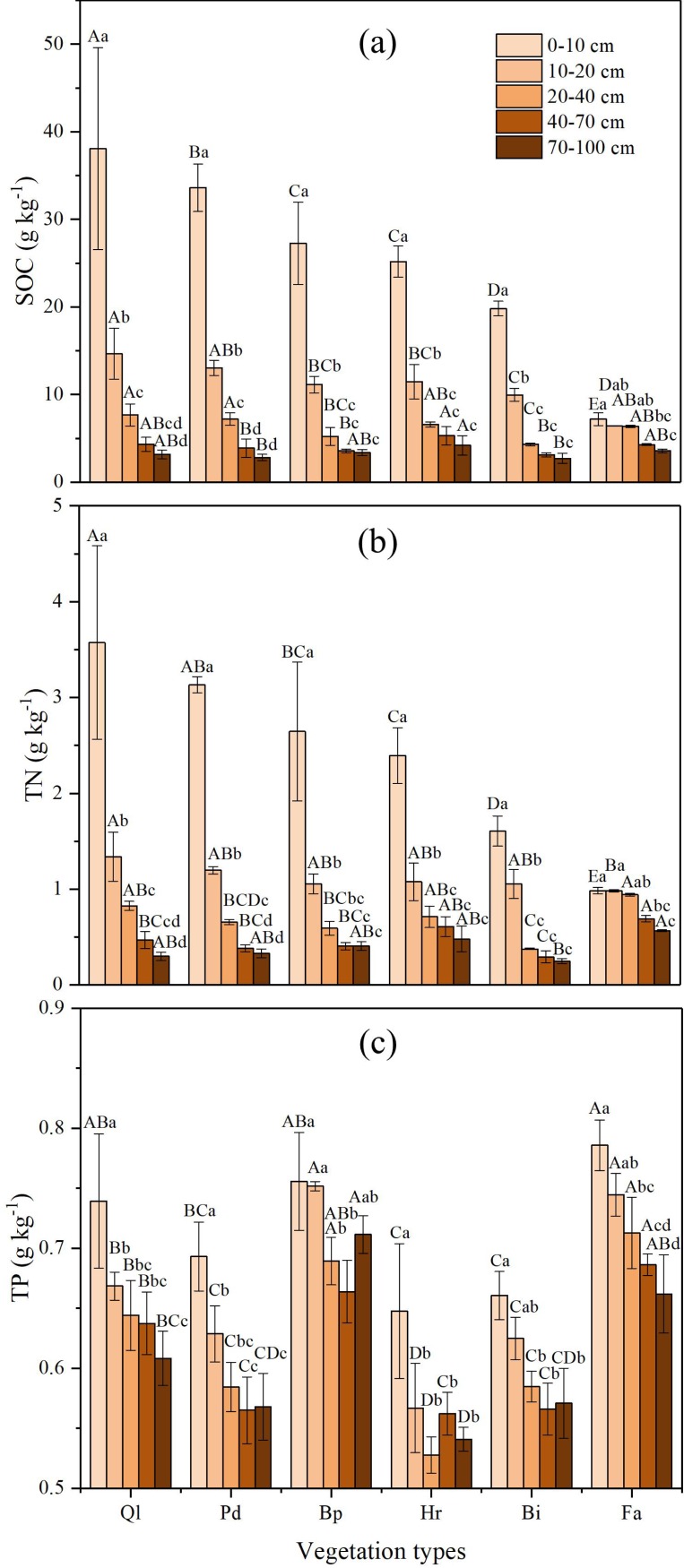
Variation of nutrient concentrations in soil profile of different vegetation types. (A) Soil organic carbon; (B) Total nitrogen; (C) Total phosphorus. Data are shown as the mean ± standard deviation. The sample size *n* = 3. Different small letters in the same vegetation type mean significant difference in different soil layers, and different capital letters in the same soil layer mean significant difference in different vegetation types at 0.05 level. Bi, Hr, Bp, Pd and Ql represent *Bothriochloa ischemum*, *Hippophae rhamnoides*, *Betula platyphylla*, *Populus davidiana*, *Pinus tabuliformis*, and *Quercus liaotungensis*, respectively.

The SOC concentrations in different soil layers increased gradually with advancing vegetation restoration (Fig. 2A). The highest SOC concentrations of all vegetation stages appeared in the 0–10 cm soil layer, which illustrates that SOC mainly accumulates in the top soil layer. The SOC concentrations of climax forest (Ql) were higher than those of other restoration stages in 0–40 cm soil layers. The highest SOC concentrations in the 40–100 cm soil layers were found in shrub (Hr), whereas the lowest appeared in grassland (Bi). Overall, the SOC concentrations in the 0–40 cm soil layers in each restoration stage varied substantially, whereas SOC concentrations in the 40–100 cm soil layers in each restoration stage exhibited no obvious changes.

The concentration of TN increased with vegetation succession (Fig. 2B) and was significantly different between different restoration stages. The highest TN concentration of all vegetation stages was detected in the 0–10 cm soil layer, displaying a similar tendency to the SOC. The concentration of TN in climax forest (Ql) were significantly higher than those of other restoration stages at soil depth of 0–20 cm. The highest TN in the 20–100 cm soil layers occurred in farmland, whereas the lowest appeared in grassland (Bi). The TN concentration in different successional stages decreased with increasing depth in the 0–100 cm soil profile, and all of them decreased significantly in the 0–40 cm soil layer. Below 40 cm, the concentration of TN showed a tendency toward stabilization.

In general, variation was small for soil TP concentrations, ranging from 0.51 to 0.82 g kg^−1^, with an average concentration of 0.64 g kg^−1^ (Fig. 2C). The TP concentration in each restoration stage decreased with an increase in soil depth in the 0–100 cm soil layer, and is distributed evenly in the soil profiles. The TP concentrations of farmland and early forest (Bp) were significantly higher than those of other vegetation types in 0–100 cm, whereas the TP concentration had not significant interaction with vegetation restoration.

Nested ANOVA indicated that both soil depth and vegetation type significantly affected SOC, TN and TP. Soil depth showed a greater effect on SOC and TN than vegetation type, whereas vegetation type had a bigger impact on TP than soil depth (Table 3).

### SOC, TN, and TP stoichiometric characteristics

#### Statistical analysis of SOC, TN, and TP stoichiometry in soil profile

The soil C:N, C:P, and N:P ratios decreased with an increase in soil depth (Table 4). The C:N ratio in the 0–10 cm soil layer was significantly higher than that in the 10–100 cm soil layers (*P* < 0.05). The C:N ratio in the 10–100 cm soil layer did not significantly vary. The variation of the C:N ratio was smaller and relatively stable compared with the SOC and TN concentrations. In contrast, the variation of the C:P ratio was large. The mean value of the C:P ratio from soil depths of 0–10 to 20–40 cm decreased from 39.18 to 10.25 (decrease of 73.84%). The soil C:P ratio in the 40–100 cm soil layer had no significant change. The N:P ratio in the 0–20 cm soil layer was significantly larger than that in the lower soil layer (*P* < 0.05). The average value of N:P ratio from soil depths of 0–10 to 20–40 cm decreased from 3.64 to 1.07 (decrease of 70.60%). However, the soil N:P ratio of the 40–100 cm layer displayed no significant changes.

**Table 3:** Nested ANOVA results for the effects of vegetation type and soil depth on SOC, TN and TP in Ziwuling forest area.

Factor	SOC		TN		TP	
	F	*P*	F	*P*	F	*P*
Soil depth	162.00	<.0001	81.70	<.0001	7.37	<.0001
Vegetation type	66.24	<.0001	21.60	<.0001	62.52	<.0001

**Table 4:** Stoichiometries of SOC, TN, and TP in soil profile. Different lowercase letters in the same C:N,C:P, and N:P ratios indicate significant difference in different soil layers at 0.05 confidence interval. The sample size *n* = 3.

	Soil depth (cm)	Maximum (g kg^−1^)	Minimum (g kg^−1^)	Mean (g kg^−1^)	Standard deviation (g kg^−1^)	Coefficient of variation
C:N	0–10	13.64	6.96	10.87a	1.57	0.15
	10–20	11.25	6.53	9.68b	1.22	0.12
	20–40	11.93	6.75	9.51b	1.53	0.16
	40–70	13.00	6.19	9.37b	1.75	0.19
	70–100	13.64	6.31	9.52b	1.89	0.20
C:P	0–10	56.76	8.35	39.18a	12.20	0.31
	10–20	25.58	8.44	18.11b	4.29	0.24
	20–40	13.51	6.50	10.25c	2.51	0.25
	40–70	11.06	4.69	6.76c	1.79	0.26
	70–100	10.01	4.35	5.60c	1.38	0.25
N:P	0–10	5.47	1.20	3.64a	1.14	0.31
	10–20	2.35	1.29	1.71b	0.31	0.18
	20–40	1.48	0.61	1.07c	0.29	0.27
	40–70	1.22	0.45	0.74c	0.23	0.30
	70–100	1.15	0.43	0.61c	0.20	0.32

### Changes of C:N, C:P, and N:P ratios in soil profiles of different vegetation types

**Figure 3:**
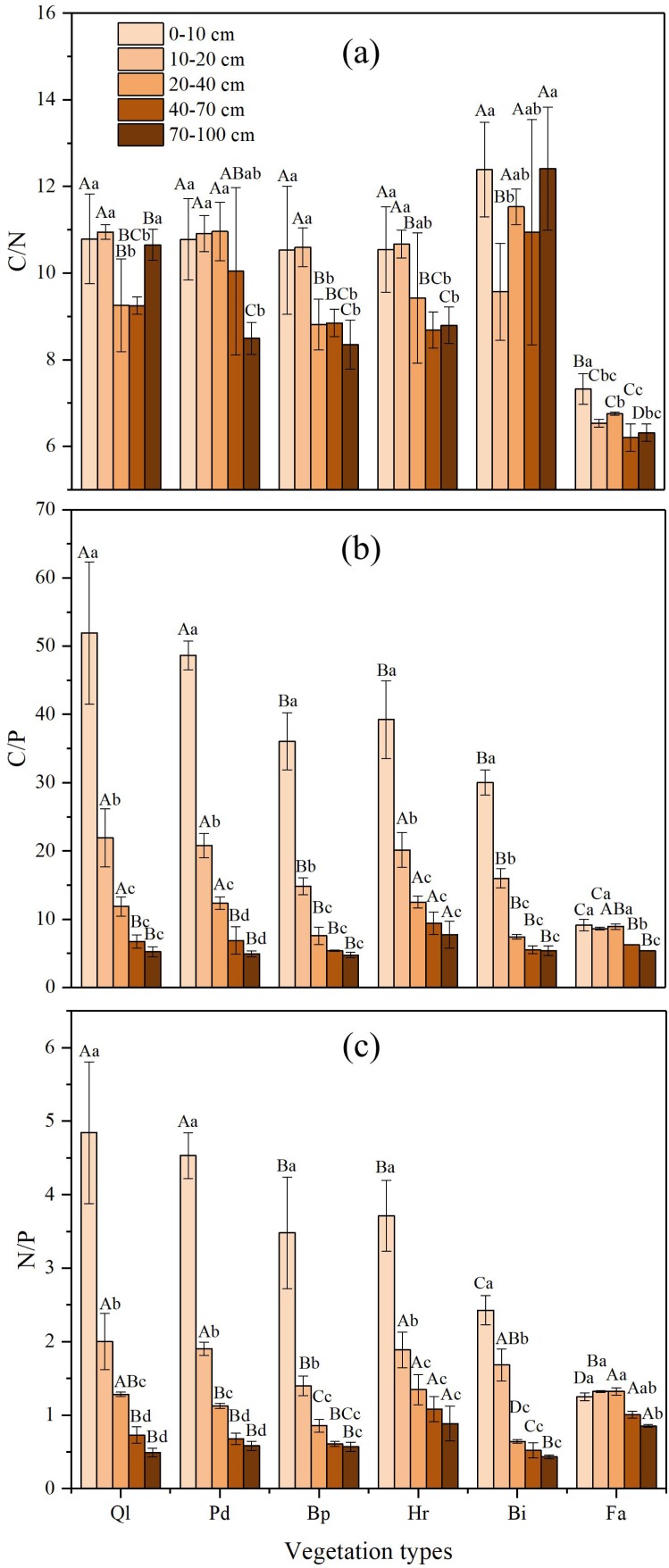
C:N, C:P and N:P ratios in soil profile of different vegetation type. (A) The ratio of soil organic carbon to total nitrogen; (B) The ratio of soil organic carbon to total phosphorus; (C) The ratio of total nitrogen to total phosphorus. Data are shown as the mean ± standard deviation. The sample size *n* = 3. Different small letters in the same vegetation type mean significant difference in different soil layers; and different capital letters in the same soil layer mean significant difference in different vegetation types at 0.05 level. Bi, Hr, Bp, Pd and Ql represent *Bothriochloa ischemum*, *Hippophae rhamnoides*, *Betula platyphylla*, *Populus davidiana*, *Pinus tabuliformis*, and *Quercus liaotungensis*, respectively.

The soil C:N ratio was relatively stable along the gradient of vegetation restoration (Fig. 3A). The highest C:N ratio in the different vegetation restoration stages appeared in grassland (Bi), whereas the lowest occurred in farmland. The soil C:N ratio of farmland was lower than those of other vegetation types, which might be the result of the high TN concentrations in farmland. The variation of the C:N ratio in 0–100 cm soil layer was small and remain stable at about 10.

In general, the soil C:P ratio increased gradually along with the progression of the vegetation succession, and significant differences were found between different restoration stages (Fig. 3B). The highest C:P ratios appeared in climax forest (Ql) and early forest (Pd), whereas the lowest occurred in farmland. Furthermore, in the 0–100 cm soil profile, the C:P ratio decreased with an increase in soil depth. Soil C:P ratio in soil depth of over 40 cm varied substantially according to restoration stage, whereas the C:P ratio in soil depth below 40 cm exhibited small variation.

The soil N:P ratio showed a similar trend to the C:P ratio (Fig. 3C). The N:P ratio in the different soil layers increased gradually alongside vegetation restoration. In the vertical direction, the N:P ratio in each restoration stage decreased with increase in soil depth in the 0–100 cm soil layer. The highest N:P ratio in different vegetation restoration stages appeared in climax forest Ql and early forest Pd, whereas the lowest occurred in farmland. Soil N:P ratio in soil depth of over 40 cm in each restoration stage varied substantially, whereas the N:P ratio in soil depth below 40 cm exhibited small variations.

Nested ANOVA indicated that both soil depth and vegetation type significantly affected C:N, C:P and N:P. Soil depth showed a greater effect on C:P and N:P than vegetation type, whereas vegetation type had a bigger impact on C:N than soil depth (Table 5).

**Table 5:** Nested ANOVA results for the effects of vegetation type and soil depth on C:N:P stoichiometric characteristics in Ziwuling forest area.

Factor	C:N		C:P		N:P	
	F	*P*	F	*P*	F	*P*
Soil depth	2.44	0.0027	85.92	<.0001	79.75	<.0001
Vegetation type	34.05	<.0001	45.71	<.0001	27.89	<.0001

### Correlations of ecological stoichiometry between SOC, TN, and TP

Soil C, N, and P showed a strong significant correlation (*P* < 0.01) (Table 6). Soil C showed a significant and positive correlation to the C:N, C:P, and N:P ratios (*P* < 0.01). Soil N was highly positively correlated to C:P, and N:P ratios (*P* < 0.01), but not correlated with C:N ratio (*P* > 0.05). Soil P was highly positively correlated to C:P and N:P ratios (*P* < 0.01). However, the correlation between P and C:N was negative (*P* > 0.05). In addition, positive correlations (*P* < 0.05) among C:N, C:P, and N:P ratios were observed.

**Table 6:** Correlation analysis between soil nutrient concentrations and ecological stoichiometry. The sample size n = 90.

Index	N	P	C:N	C:P	N:P
C	0.966^**^	0.241^**^	0.325^**^	0.992^**^	0.984^**^
N	1	0.290^**^	0.190	0.965^**^	0.991^**^
P		1	−0.160	0.402^**^	0.449^**^
C:N			1	0.251^**^	0.235^*^
C:P				1	0.965^**^

**Notes.**

* *p* < 0.05.

** *p* < 0.01.

## Discussion

### Response of soil nutrient concentrations to vegetation succession and soil depth

C, N, and P in soil are important for maintaining sustainable and productive ecosystems (Agren, 2008; Laik et al., 2009; Mcgroddy, Daufresne & Hedin, 2004). The average concentrations of C, N, and P in the study area were 27.20, 2.39, and 0.71 g kg^−1^, respectively. The average concentration of soil C was close to that of the global (25.71 g kg^−1^) (Cleveland & Liptzin, 2007) and national (29.51 g kg^−1^) (Tian et al., 2010) average. The average concentration of soil N was higher than that of the global (2.10 g kg^−1^) (Cleveland & Liptzin, 2007) and national (2.30 g kg^−1^) values (Tian et al., 2010). The average concentration of soil P was also higher than that of the national average (0.56 g kg^−1^) (Tian et al., 2010). Compared to other findings on the change of soil nutrient concentrations after vegetation restoration (Jiao et al., 2013; Zhang et al., 2019), the overall concentration of soil nutrient in Ziwuling forest area was also higher. This result indicates that the concentration of C in the Ziwuling forest area was relatively high, N and P nutrient elements were abundant, and vegetation restoration increases the soil nutrient concentration and quality in the region to an extent.

Long-term natural vegetation restoration could have strong effects on soil quality and the carbon and nitrogen cycle (Grünzweig et al., 2007; Vandenbygaart et al., 2011). In this study, vegetation restoration significantly increased the concentrations of SOC and TN. The results are in agreement with those in previous studies (Fu et al., 2010; Yang & Luo, 2011; Zhang et al., 2019; Zhao et al., 2017). These results were due to the ability for long-term natural vegetation restoration to improve vegetation primary productivity, thereby resulting in the improvement of the quantity and quality of litter and rhizodeposition (Cao & Chen, 2017; Deng et al., 2016; Li et al., 2016), and ultimately releasing a large amount of organic C and N into the soil. Meanwhile, soil temperature and moisture may change during the process of vegetation restoration, which in turn could lead to the enhancement of soil microbial and enzymatic activities and increased nutrient decomposition rate (Gispert et al., 2013; Zhang et al., 2019), thereby increasing the soil C and N concentrations. Compared with the soil C and N, soil P did not significantly change with vegetation restoration. Soil P is mainly affected by parent material, vegetation type, land use, and biogeochemical processes in the soil (Kooijman, Jongejans & Sevink, 2005; Lane, Noske & Sheridan, 2011). As the climate and parent material is similar across all vegetation types, the variation in soil P is not as obvious as that of C and N. The soil nutrient concentrations of different vegetation types were significantly different. The C and N concentrations were highest in forest (Ql) and lowest in farmland. Increased C and N concentrations in forests may be due to the accumulation of surface leaves and litter, whereas crop harvests on farmlands decrease the return of nutrients to the soil. This indicates that reasonable agricultural management practices, such as retaining manure and crop residues, are vital to maintaining and improving soil fertility (Kirkby et al., 2013).

The depth of sampling is an important factor for predicting spatial variation of soil nutrients (Vandenbygaart et al., 2011). Our results found that the concentrations of C, N, and P in the study area decreased with an increase in soil depth, which is consistent with most existing studies (Cao & Chen, 2017; Xu et al., 2019; Zhang & Shangguan, 2018). At the same time, changes in C and N concentrations in the surface soil were clearly observed across the progression of vegetation succession, which is also supported by previous research (Tian et al., 2010; Xu et al., 2019). Soil nutrient is influenced not only by soil parent materials, but also by litter decomposition, root architecture, and exudates (Berger, Neubauer & Glatzel, 2002; Clemmensen et al., 2013; Gao et al., 2014; Xu et al., 2019). Microorganism-driven litter decomposition mainly occurs in surface soil (0–20 cm) (Gao et al., 2014), which increases the nutrient concentration in surface soil. With an increase of soil depth, the input of organic matter decreases due to the decrease of microbial decomposition activity and root exudates (Berger, Neubauer & Glatzel, 2002; Clemmensen et al., 2013). Deng et al. (2018) found that the amount of litter and roots in surface soil (0–20 cm) in different vegetation succession stages was significantly higher than that of subsoil (20–60 cm), which had a significant positive effect on SOC and soil C sequestration. P is mainly affected by soil parent material, which is a sedimentary mineral with poor migration in soil (Ren et al., 2016; Walker & Syers, 1976; Wang, Zhang & Haung, 2009), and therefore the distribution of soil P in the vertical profile is more uniform.

### Responses of soil stoichiometry to vegetation succession and soil depth

Long-term vegetation restoration from farmland to forest had very significant effects on soil nutrient composition. The variation of SOC, TN, and TP concentrations in soil caused changes in nutrient stoichiometric relations (Agren, 2008; Tian et al., 2010). The soil C:N ratio is a measure of C and N nutrient balance, which affects the cycling of organic C and N, and is a sensitive indicator of soil quality (Tian et al., 2010). The average soil C:N ratio in this study was 10.87, which was lower than that of the average Chinese soil (12.30) and the average global forest soil (12.40) (Mcgroddy, Daufresne & Hedin, 2004; Tian et al., 2010). Other studies showed that the soil C:N ratio was inversely proportional to the decomposition rate of organic matter, and soil with a low C:N ratio has a faster mineralization rate (Cleveland & Liptzin, 2007; Wang & Yu, 2008), indicating that the soil C accumulation, organic matter decomposition and mineralization rate of the Ziwuling forest area would be faster than average. The C:N ratio in the soil also remained remarkably stable along the process of vegetation restoration, which is in line with the results reported for secondary forests worldwide (Yang & Luo, 2011; Zinn, Marrenjo & Silva, 2018). This is due to C and N, as structural components, have relatively fixed ratios in the process of accumulation and consumption (Cleveland & Liptzin, 2007). Zinn, Marrenjo & Silva (2018) also discovered that soil C:N ratios are unresponsive to land use change. Furthermore, soil C:N ratios were stable across the soil profile across different vegetation types, which is consistent with previous reports (Tian et al., 2010; Wang et al., 2014). This is due to the close temporal coupling of C and N concentrations in litter and roots (Fan et al., 2015; Mcgroddy, Daufresne & Hedin, 2004; Yang & Luo, 2011).

In general, the soil C:P ratio is considered a marker of soil P mineralization. It is also an index that is used to measure the microbial mineralization of soil organic matter to release or absorb potential P from the environment (Tian et al., 2010). A low C:P ratio is conducive to the release of nutrients by microorganisms via the process of organic matter decomposition and promotes the increase of effective P in the soil. On the contrary, a high C:P ratio leads to the limitation of P resulting from the decomposition of organic matter by microorganisms, microorganisms will compete with the plant for soil inorganic P, that is not conducive for plant growth (Wang et al., 2014). The average value of the soil C:P ratio in this study was 39.18, which was lower than that of the average for China soil (52.70) and global forest soil (81.90) (Mcgroddy, Daufresne & Hedin, 2004; Tian et al., 2010). This value shows that the availability of P in the Ziwuling forest area is relatively high. In addition, SOC and TN were significantly correlated, and the soil C:P and soil N:P had a similar trend in response to vegetation restoration. The soil C:P ratio increased with vegetation succession. Compared across different vegetation types, the C:P ratio was highest in climax forest (Ql) and early forest (Pd). These results suggest that P would be a limiting element for plant growth during vegetation succession. As P mainly comes from weathering and leaching of rocks and showed low biological availability in the Loess Plateau (Wang, Zhang & Haung, 2009), the uptake of P by plants gradually increasing during vegetation succession (Ren et al., 2016) results in P becoming increasingly limited. Previous studies also show that a P limitation is exacerbated during forest succession (Fan et al., 2015; Huang et al., 2013; Ren et al., 2016).

Soil N:P ratio can serve as an indicator of N saturation, which in turn indicates the availability of soil nutrient elements during plant growth and is used to determine the threshold of nutrient restriction (Güsewell, Koerselman & Verhoeven, 2003; Tessier & Raynal, 2003). The average soil N:P ratio in the Ziwuling forest area was 3.62, which was lower than that of China’s terrestrial soil with an average of 3.9 (Tian et al., 2010), but higher than those found by other scholars on the Loess Plateau (Bai et al., 2019; Cao & Chen, 2017). With the restoration of vegetation, the concentration of N in soil increased significantly, whereas that of P remained stable, resulting in an increasing soil N:P ratio. Moreover, some studies have shown that soil N:P ratio was negatively correlated with plant growth rate (Fan et al., 2015). The N:P ratio increases at low plant growth rates and decreases at high growth rates. In this study, the soil N:P ratio was lower in the early successional stage and higher in the later stages. In other words, plant growth may be subject to nitrogen limitation in the early stages of vegetation succession. Previous studies have also shown that nutrient limitation changed with vegetation succession in the development of soil ecosystem, transitioning from N limitation in the early successional stage to P limitation in the late successional stage (Reed & Cleveland, 2011; Hayes et al., 2014). The total amount of N in the soil decreased with an increase in soil depth, and the relative stability of TP concentration at the profile level led to a decrease of the N:P ratio with an increase in soil depth.

In summary, C:N:P stoichiometry plays a key role in the structure and function of ecosystems (Agren, 2008; Ladanai, Agren & Olsson, 2010). In this study, we only explored the soil C:N:P stoichiometry across vegetation types and age sequence on the Loess Plateau. However, the above-and below-ground ecosystem processes are closely integrated, and their interactions have an important effect on ecosystem processes and properties (Wardle et al., 2004). Therefore, further research on the C:N:P stoichiometry relationships between plants and soils at different scales, as well as effects of vegetation succession on soil physical and biological properties, and their relationship with soil nutrient concentration is necessary. These results can inform management practices for forest policymakers aiming to create sustainable forest ecosystems, particularly for the large-scale natural secondary forest on the Loess Plateau, China.

## Conclusions

We investigated the changes of soil nutrient concentrations and C:N:P stoichiometry in 0–100 cm soil profile following vegetation restoration in the Loess Plateau Region. Our study suggested that long-term vegetation restoration can enhance the concentrations of SOC and TN and ratios of soil C:P and N:P, whereas the concentrations of soil TP and the ratio of C:N did not improve substantially with vegetation restoration. The concentrations of SOC and TN and the ratios of C:P and N:P decreased with an increase of soil depth (0–100 cm), and they were most prone to change in surface soil (0–20 cm). Furthermore, our study indicated that in the early stage of succession for degraded grasslands, adding N fertilization may enhance the growth of plants, and increasing P application can avoid P limitation during the later recovery period.

##  Supplemental Information

10.7717/peerj.8382/supp-1 Data S1 Raw data: Soil nutrient concentration

## Additional Information and Declarations

### Field Study Permissions

The following information was supplied relating to field study approvals (i.e., approving body and any reference numbers): The research station is called Lianjiabian forest farm and is managed by Ziwuling Forestry Administration of Heshui County, Gansu Province. We collected very few samples in the research area, so it would not cause damage to the local environment. When taking samples, we obtained oral approval from the head of Ziwuling Forestry Administration, Wangji Fan, and took samples with the help of the staff.

### Data Availability

The following information was supplied regarding data availability: The raw measurements are available in the Supplemental File.

## References

[ref-1] Agren GI (2008). Stoichiometry and nutrition of plant growth in natural communities. Annual Review of Ecology Evolution and Systematics.

[ref-2] Bai X, Wang B, An S, Zeng Q, Zhang H (2019). Response of forest species to C:N:P in the plant-litter-soil system and stoichiometric homeostasis of plant tissues during afforestation on the Loess Plateau, China. Catena.

[ref-3] Berger TW, Neubauer C, Glatzel G (2002). Factors controlling soil carbon and nitrogen stores in pure stands of Norway spruce (*Picea abies*) and mixed species stands in Austria. Forest Ecology and Management.

[ref-4] Cao Y, Chen Y (2017). Coupling of plant and soil C:N:P stoichiometry in black locust (*Robinia pseudoacacia*) plantation on the Loess Plateau, China. Trees.

[ref-5] Cheng J, Cheng J, Shao H, Zhao L, Yang X (2012). Soil seed banks and forest succession direction reflect soil quality in Ziwuling Mountain, Loess Plateau, China. Clean-Soil Air Water.

[ref-6] Clemmensen KE, Bahr A, Ovaskainen O, Dahlberg A, Ekblad A, Wallander H, Stenlid J, Finlay RD, Wardle DA, Lindahl BD (2013). Roots and associated fungi drive long-term carbon sequestration in Boreal forest. Science.

[ref-7] Cleveland CC, Liptzin D (2007). C:N:P stoichiometry in soil: is there a “Redfield ratio” for the microbial biomass?. Biogeochemistry.

[ref-8] Deng J, Sun P, Zhao F, Han X, Yang G, Feng Y, Ren G (2016). Soil C, N, P and its stratification ratio affected by artificial vegetation in subsoil, Loess Plateau China. PLOS ONE.

[ref-9] Deng L, Wang K, Zhu G, Liu Y, Chen L, Shangguan Z (2018). Changes of soil carbon in five land use stages following 10 years of vegetation succession on the Loess Plateau, China. Catena.

[ref-10] Fan H, Wu J, Liu W, Yuan Y, Hu L, Cai Q (2015). Linkages of plant and soil C:N:P stoichiometry and their relationships to forest growth in subtropical plantations. Plant and Soil.

[ref-11] Fu B, Chen L, Ma K, Zhou H, Wang J (2000). The relationships between land use and soil conditions in the hilly area of the loess plateau in northern Shaanxi, China. Catena.

[ref-12] Fu X, Shao M, Wei X, Robertm H (2010). Soil organic carbon and total nitrogen as affected by vegetation types in Northern Loess Plateau of China. Geoderma.

[ref-13] Gao Y, He N, Yu G, Chen W, Wang Q (2014). Long-term effects of different land use types on C, N, and P stoichiometry and storage in subtropical ecosystems: a case study in China. Ecological Engineering.

[ref-14] Gispert M, Emran M, Pardini G, Doni S, Ceccanti B (2013). The impact of land management and abandonment on soil enzymatic activity, glomalin content and aggregate stability. Geoderma.

[ref-15] Grünzweig JM, Gelfand I, Fried Y, Yakir D (2007). Biogeochemical factors contributing to enhanced carbon storage following afforestation of a semi-arid shrubland. Biogeosciences.

[ref-16] Güsewell S, Koerselman W, Verhoeven JTA (2003). Biomass n:p ratios as indicators of nutrient limitation for plant populations in wetlands. Ecological Applications.

[ref-17] Han W, Fang J, Guo D, Zhang Y (2005). Leaf nitrogen and phosphorus stoichiometry across 753 terrestrial plant species in China. New Phytologist.

[ref-18] Hayes P, Turner BL, Lambers H, Laliberte E, Bellingham P (2014). Foliar nutrient concentrations and resorption efficiency in plants of contrasting nutrient-acquisition strategies along a 2-million-year dune chronosequence. Journal of Ecology.

[ref-19] Huang W, Liu J, Wang Y, Zhou G, Han T, Li Y (2013). Increasing phosphorus limitation along three successional forests in southern China. Plant and Soil.

[ref-20] Jia G, Cao J, Wang C, Wang G (2005). Microbial biomass and nutrients in soil at the different stages of secondary forest succession in Ziwuling, northwest China. Forest Ecology and Managemet.

[ref-21] Jiao F, Wen Z, An S, Yuan Z (2013). Successional changes in soil stoichiometry after land abandonment in Loess Plateau, China. Ecological Engineering.

[ref-22] Kirkby CA, Richardson AE, Wade GJ, Batten D, Blanchard C, Kirkegaard JA (2013). Carbon-nutrient stoichiometry to increase soil carbon sequestration. Soil Biology and Biochemistry.

[ref-23] Kooijman AM, Jongejans J, Sevink J (2005). Parent material effects on Mediterranean woodland ecosystems in NE Spain. Catena.

[ref-24] Ladanai S, Agren GI, Olsson BA (2010). Relationships between tree and soil properties in *Picea abies* and *Pinus sylvestris* forests in Sweden. Ecosystems.

[ref-25] Laik R, Kumar K, Das DK, Chaturvedi OP (2009). Labile soil organic matter pools in a calciorthent after 18 years of afforestation by different plantations. Applied Soil Ecology.

[ref-26] Lane PNJ, Noske PJ, Sheridan GJ (2011). Phosphorus enrichment from point to catchment scale following fire in *eucalypt* forests. Catena.

[ref-27] Li J, Liu Y, Hai X, Shangguan Z, Deng L (2019). Dynamics of soil microbial C:N:P stoichiometry and its driving mechanisms following natural vegetation restoration after farmland abandonment. Science of the Total Environment.

[ref-28] Li C, Zhao L, Sun P, Zhao F, Kang D, Yang G, Han X, Feng Y, Ren G (2016). Deep soil C, N, and P stocks and stoichiometry in response to land use patterns in the loess hilly region of China. PLOS ONE.

[ref-29] Ma W, Li J, Jimoh SO, Zhang Y, Guo F, Ding Y, Li X, Hou X (2019). Stoichiometric ratios support plant adaption to grazing moderated by soil nutrients and root enzymes. PeerJ.

[ref-30] Manzoni S, Porporato A (2009). Soil carbon and nitrogen mineralization: theory and models across scales. Soil Biology and Biochemistry.

[ref-31] Mcgroddy ME, Daufresne T, Hedin LO (2004). Scaling of C:N:P stoichiometry in forest worldwide: implications of terrestrial Redfield-type ratios. Ecology.

[ref-32] Nelson DW, Sommers LE (1982). Methods of soil analysis. Part 2 chemical and microbiological properties.

[ref-33] Parkinson J, Allen S (1975). A wet oxidation procedure suitable for the determination of nitrogen and mineral nutrients in biological material. Communications in Soil Science and Plant Analysis.

[ref-34] Reed SC, Cleveland VCC (2011). Are patterns in nutrient limitation belowground consistent with those aboveground: results from a 4 million year chronosequence. Biogeochemistry.

[ref-35] Ren C, Zhao F, Kang D, Yang G, Han X, Tong X, Feng Y, Ren G (2016). Linkages of C:N:P stoichiometry and bacterial community in soil following afforestation of former farmland. Forest Ecology and Management.

[ref-36]  Sparks DL, Page A, Helmke P, Loeppert R, Soltanpour P, Tabatabai M, Johnston C, Sumner M (1996). Methods of soil analysis. Part 3-chemical methods.

[ref-37] Tessier JT, Raynal DJ (2003). Use of nitrogen to phosphorus ratios in plant tissue as an indicator of nutrient limitation and nitrogen saturation. Journal of Applied Ecology.

[ref-38] Tian H, Chen G, Zhang C, Melillo JM, Hall CAS (2010). Pattern and variation of C:N:P ratios in China’s soils: a synthesis of observational data. Biogeochemistry.

[ref-39] Vandenbygaart AJ, Bremer E, Mcconkey BG, Ellert BH, Janzen HH, Angers DA, Carterf MR, Druryg CF, Lafondh GP, McKenzie RH (2011). Impact of sampling depth on differences in soil carbon stocks in long-term agroecosystem experiments. Soil Science Society of America Journal.

[ref-40] Walker TW, Syers JK (1976). The fate of phosphorus during pedogenesis. Geoderma.

[ref-41] Wang S, Yu G (2008). Ecological stoichiometry characteristics of ecosystem carbon, nitrogen and phosphorus elements. Acta Ecologica Sinica.

[ref-42] Wang W, Sardans J, Zeng C, Zhong C, Li Y, Peñuelas J (2014). Responses of soil nutrient concentrations and stoichiometry to different human land uses in a subtropical tidal wetland. Geoderma.

[ref-43] Wang Y, Zhang X, Haung C (2009). Spatial variability of soil total nitrogen and soil total phosphorus under different land uses in a small watershed on the Loess Plateau. China Geoderma.

[ref-44] Wardle DA, Bardgett RD, Klironomos JN, Setala H, Putten van der WH, Wall DH (2004). Ecological linkages between aboveground and belowground biota. Science.

[ref-45] Wei X, Shao M, Fu X, Horton R, Li R, Zhang X (2009). Distribution of soil organic C, N and P in three adjacent land use patterns in the northern Loess Plateau, China. Biogeochemistry.

[ref-46] Xu H, Qu Q, Li P, Guo Z, Wu L, Wulan E, Xu X (2019). Stocks and stoichiometry of soil organic carbon, total nitrogen, and total phosphorus after vegetation restoration in the Loess Hilly Region, China. Forests.

[ref-47] Yang W, Cheng H, Hao F, Ouyang W, Liu S, Lin C (2012). The influence of land-use change on the forms of phosphorus in soil profiles from the Sanjiang Plain of China. Geoderma.

[ref-48] Yang Y, Liu B, An S (2018). Ecological stoichiometry in leaves, roots, litters and soil among different plant communities in a desertified region of Northern China. Catena.

[ref-49] Yang Y, Luo Y (2011). Carbon: nitrogen stoichiometry in forest ecosystems during stand development. Global Ecology and Biogeography.

[ref-50] Zhang W, Liu W, Xu M, Deng J, Han X, Yang G, Feng Y, Ren G (2019). Response of forest growth to C:N:P stoichiometry in plants and soils during *Robinia pseudoacacia* afforestation on the Loess Plateau, China. Geoderma.

[ref-51] Zhang Y, Shangguan Z (2018). Interaction of soil water storage and stoichiometrical characteristics in the long-term natural vegetation restoration on the Loess Plateau. Ecological Engineering.

[ref-52] Zhao F, Kang D, Han X, Yang G, Feng Y, Ren G (2015). Soil stoichiometry and carbon storage in long-term afforestation soil affected by understory vegetation diversity. Ecological Engineering.

[ref-53] Zhao F, Zhang L, Sun J, Ren CJ, Han XH, Peng G, Bai H (2017). Effect of soil C, N and P stoichiometry on soil organic C fractions after afforestation. Pedosphere.

[ref-54] Zinn YL, Marrenjo GJ, Silva CA (2018). Soil C:N ratios are unresponsive to land use change in Brazil: a comparative analysis. Agriculture Ecosystems and Environment.

